# New clinical application of magnetocardiography: diagnosis of left ventricular hypertrophy

**DOI:** 10.3389/fcvm.2025.1577662

**Published:** 2025-05-27

**Authors:** Xiaoxia Chen, Li Li, Xuanhao Xu, Lei Xia, Chao Liu, Chengxing Shen, Yukun Luo

**Affiliations:** ^1^ Department of Cardiology, Fujian Medical University Union Hospital, Fuzhou, Fujian, China; ^2^ General Medicine Department, Fuzhou NO. 1 Hospital Affiliated with Fujian Medical University, Fuzhou, Fujian, China; ^3^ Department of Cardiology, Shanghai Sixth People’s Hospital Affiliated to Shanghai Jiao Tong University School of Medicine, Shanghai, China; ^4^ Joint Laboratory of Bioimaging Technology and Applications, SAS-SIMIT & MEDI, Shanghai, China; ^5^ Department of Emergency, Fujian Medical University Union Hospital, Fuzhou, Fujian, China

**Keywords:** magnetocardiography (MCG), left ventricular hypertrophy (LVH), echocardiography, application, diagnosis

## Abstract

**Introduction:**

Magnetocardiography (MCG), as a high-sensitivity, non-contact and non-invasive technology, is assumed to detect weak electrophysiological changes. MCG can detect early signs of myocardial ischemia that may be missed by other diagnostic tools. Present research aims to evaluate the possibility of using MCG to detect left ventricular hypertrophy (LVH).

**Methods:**

The study included 481 participants, categorized into two groups according to echocardiographic criteria: the LVH group and the non-LVH group. We evaluated the performance of the MCG-derived diagnostic model for detecting LVH in the retrospective cohort and performed external validation in the prospective cohort. Using echocardiography as the reference standard, we assessed the model's area under the curve (AUC) value, sensitivity, specificity, positive predictive value (PPV), negative predictive value (NPV), and accuracy.

**Results:**

MCG parameters (S_B5_ + R_D3_, QRS area, QRS-T area, DM_50_, TT_MA) were incorporated into a logistic regression model, which produced an AUC of 0.844 (95% CI: 0.792–0.895) for the retrospective cohort. The sensitivity, specificity, PPV, NPV, and accuracy were 72.2%, 85.8%, 67.3%, 88.4%, and 81.9%. In the prospective cohort, the model exhibited an AUC of 0.782 (95% CI: 0.697–0.866), and the sensitivity, specificity, PPV, NPV, and accuracy were 63.6%, 86.5%, 74.5%, 79.4%, and 77.8%, respectively.

**Conclusion:**

Besides myocardial ischemia, MCG also provides useful information suggestive of LVH.

## Introduction

Magnetocardiography (MCG) is an advanced diagnostic technique that captures the magnetic field generated by the heart's electrical activity (1–3). MCG offers a non-contact method of recording the magnetic signals emanating from the cardiac electrophysiological processes (4, 5). Superconducting quantum interference device (SQUID) and Optically Pumped Magnetometers (OPMs) are the two mainstream technologies in MCG detecting. Although OPMs can operate at room temperature and are portable, they are more susceptible to environmental interference. In contrast, SQUID sensors offer higher sensitivity, greater stability, and clearer signals, particularly in clinical environments with stringent precision requirements (6). A remarkable advantage of MCG is that the recording of cardiac magnetic fields is not considerably impacted by the varying conductivities and electrical resistances of the diverse tissues and skins. Due to its high sensitivity, non-contact, and non-invasive, MCG has emerged as a burgeoning focus in the domain of heart disease detection.

Over the past decades, the main medical applications of the MCG are coronary artery diseases (7–9), arrhythmias (10–12) and fetal congenital heart diseases (13). Advancements in magnetic sensor technology, active shielding, denoising algorithms, and MCG computational methods are paving the way for innovative solutions that enhance the reliability of unshielded MCG measurements. These developments open up the potential to create dependable, portable devices that are more finely tuned to specific clinical needs. By seamlessly integrating with other non-invasive imaging modalities and, when necessary, electrocardiographic recordings, these instruments can offer more precise diagnostic capabilities and cater to a broader range of medical applications. MCG can detect early signs of myocardial ischemia that may be missed by other diagnostic tools. Recently,studies are ongoing to test the clinical application of MCG in the field of hypertrophic cardiomyopathy (14), inflammatory cardiomyopathy (15) and myocarditis (16).

Left ventricular hypertrophy (LVH) is characterized by the thickening of the left ventricular walls and a rise in myocardial mass (17). LVH is a substantial standalone risk factor, impacting not merely the incidence and fatality rates of cardiovascular diseases (CVDs), but also contributing to overall mortality (18–20). The incidence of LVH in adults is about 10% to 20% (21, 22). The etiology of LVH is complex, such as hypertension, intense exercise, hypertrophic cardiomyopathy (HCM), cardiac amyloidosis, etc. (23).

The electrophysiological alterations associated with LVH are predominantly manifested in the elevation of QRS amplitude and prolongation of its duration, shifts in the instantaneous and mean QRS vectors, irregularities in the ST segment and T waves, and anomalies in the *P* wave. Previous studies (24–27) reported the clinical value of MCG on detecting LVH, these studies suffered limitations including small sample sizes, and single disease type. Moreover, these studies only used a limited set of MCG parameters for LVH, resulting in low specificity and sensitivity in LVH diagnosis.

As MCG technology advanced, a variety of innovative magnetocardiography interpretation techniques have emerged, such as magnetic field mapping (MFM) and pseudo current density mapping. These developments have given rise to novel parameters, enhancing diagnostic accuracy. Nonetheless, existing research primarily utilized individual parameters (24–27), and no holistic diagnostic model has yet been established. Present research aims to validate the clinical value of LVH detection by MCG and establish an optimal MCG diagnostic model for LVH based on enriched MCG parameters.

## Methods

### Study design and population

**Figure 1:**
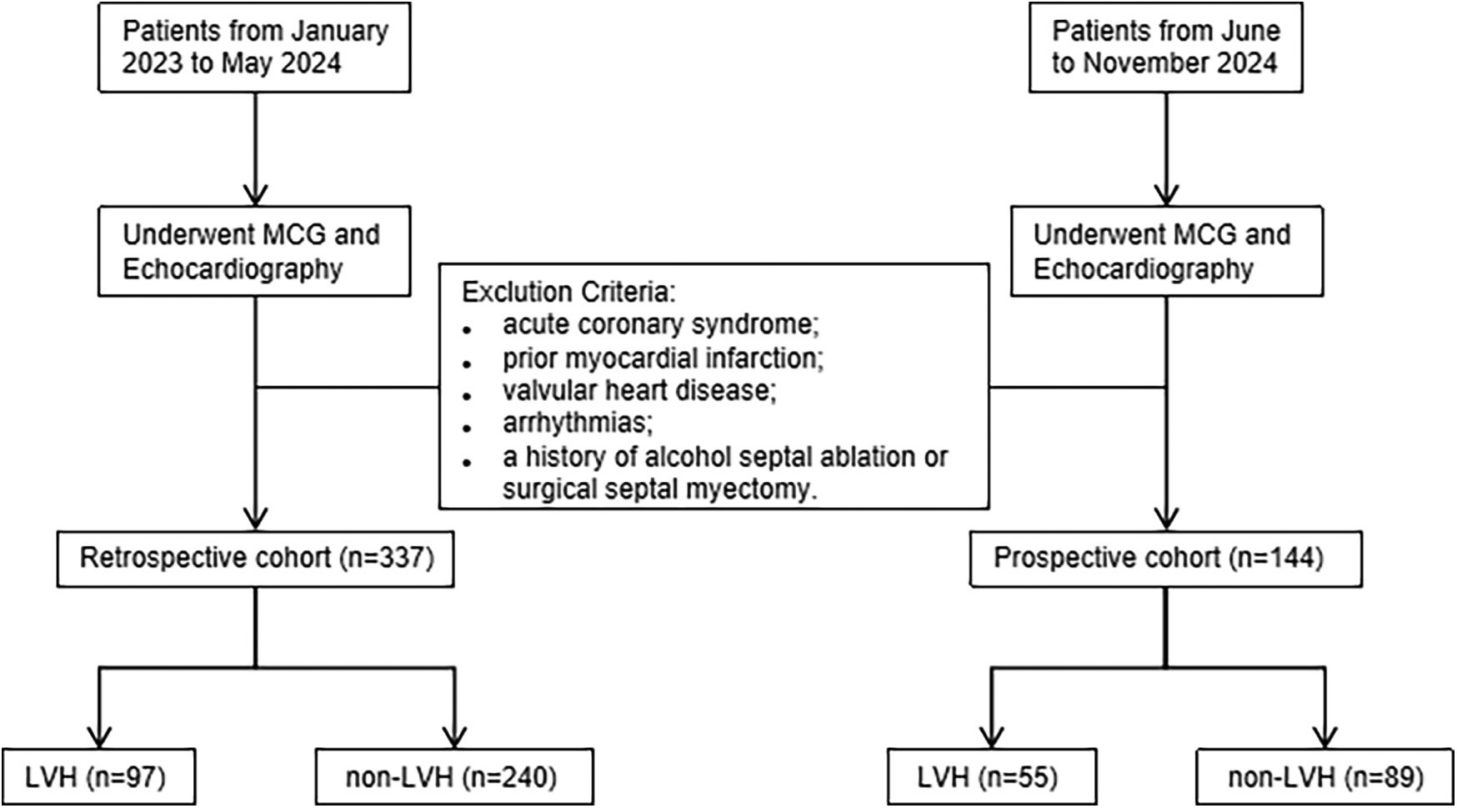
Flowchart of the study.

This single-center study utilized a cross-sectional design, including participants from both a retrospective cohort and a prospective cohort. A total of 481 patients admitted to the cardiology department of Shanghai Sixth People's Hospital were enrolled in sequence, and all of them underwent both echocardiography and MCG examination during the diagnosis and treatment process. The retrospective cohort included 97 echocardiographic confirmed LVH patients and 240 normal subjects between January 2023 and May 2024. The prospective cohort recruited 144 participants between June and November 2024 to verify the performances of the MCG diagnosis model for LVH. Subjects with acute coronary syndrome, valvular heart disease, previous myocardial infarction and arrhythmias such as ventricular premature contraction, atrial fibrillation and bundle branch block were excluded from the study. Additionally, patients who had alcohol septal ablation or surgical excision of the septum for hypertrophic cardiomyopathy were excluded. Participants with limited MCG data quality were also excluded. The flowchart illustrating our study is shown in Figure 1.

Echocardiography served as the reference standard for diagnosing LVH. According to the American Society of Echocardiography (ASE) guidelines, patients with left ventricular mass index (LVMI) ≥ 115 g/m^2^ in men and ≥95 g/m^2^ in women, or left ventricular wall thickness (LVWT) ≥ 12 mm, were categorized into the LVH group. The remaining patients were placed in the non-LVH group (28).

The protocol of the research was approved by the Hospital Ethics Committee and was duly registered with the Chinese Clinical Trial Registry under the identifier ChiCTR2000037700. Before the subjects participated, we fully informed them of the study's aims and got their written consent for inclusion in the trial.

### Echocardiography

Echocardiography was performed on the patients using the Vingmed System 7 ultrasound machine (Philips, Germany). Echocardiographic assessments were conducted by an experienced sonographer blinded to other clinical information, ensuring the objectivity of the measurements. Key parameters including left ventricular internal diameter (LVID), posterior wall thickness (PWT), and interventricular septum (IVS) were meticulously recorded. The formula endorsed by ASE was used to subsequently calculate the left ventricular mass (LVM): LVM = 0.8 * 1.04 * [(IVS + LVID + PWT)^3^ − LVID^3^] + 0.6 g. The determined LVM was subsequently normalized by indexing it to the body surface area (BSA), yielding LVMI.

### Electrocardiogram (ECG)

We captured standard 12-lead ECGs at a calibration setting of 10 mm/mV and a paper speed of 25 mm/s and a reader unaware of the other imaging data of the patients was fetched to analyze these ECGs. We measured the amplitudes of the R and S waves and the duration of the QRS complexes meticulously. By the 2018 ESC/ESH guidelines for managing arterial hypertension (29), the ECG criteria for diagnosing LVH include (1) S_V1_ + R_V5_ or R_V6_ (Sokolow-Lyon voltage) >35 mm, (2) R wave in aVL ≥11 mm, (3) S_V3_ + R_aVL_ (Cornell voltage) >28 mm for men and >20 mm for women, and (4) Cornell product (Cornell voltage * QRS duration) >2440 mm*ms. We assessed all four distinct criteria, and patients meeting any single criterion were classified as having LVH.

### MCG

**Figure 2:**
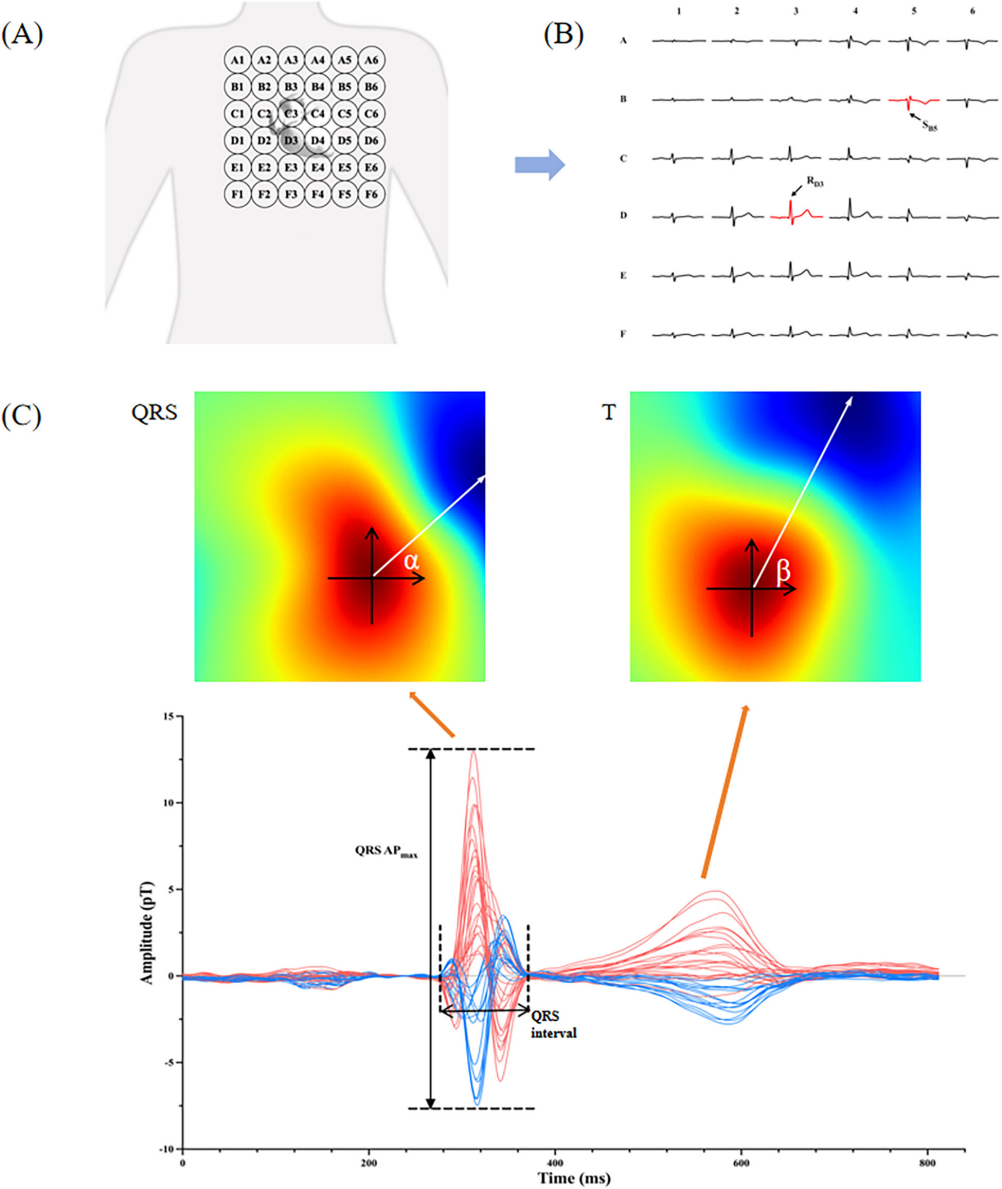
Interpretation of MCG parameters. **(A)** The probing scope of MCG sensors over the precordial area; **(B)** typical waves of 36 MCG channels detected from a healthy man, and the arrows point to the channel B5 and D3; **(C)** the lower picture is the time curve of magnetic intensity from a healthy man, and the upper pictures are the magnetic field maps (MFM) for the QRS complex and the T wave. QRS AP_max_ and QRS interval are marked in the bottom map. The angle α and β in the MFM are the averaged magnetic angle of QRS and T waves respectively, and the difference between them is QRS-T angle.

All participants underwent the MCG examinations utilizing an unshielded 9-channel MCG system (MD-U0–92001, Shanghai MEDI Instruments Ltd., Shanghai, China). The MCG system was equipped with nine SQUID sensors, spaced 4 cm apart and connected to second-order axial gradiometers. These sensors captured data from a total of 36 points positioned directly over the precordial area with the patient lying supine in a resting position. After removing any metallic or electronic interference, the MCG recordings were performed for approximately 5 min at 1,000 Hz sampling frequency. The original signals were then automatically filtered, baseline corrected, and averaged for subsequent detailed analysis. Since these MCG waveforms have a similar morphology as ECG, we adopted the same naming convention used in ECG, i.e., *P*, QRS, and *T* waves. Subsequently, the software plotted the magnetic field map (MFM) and pseudo current density mapping on the detection plane based on the averaged waves. Our study extracted a total of 10 MCG parameters to evaluate their effectiveness in detecting LVH, which were listed as the following (some as shown in Figure 2):

**S_B5_** **+** **R_D3_:** We proposed using S_B5_ + R_D3_ in MCG to evaluate the QRS amplitude, which referred to the negative amplitude of the second row, fifth column channel plus the positive amplitude of the fourth row, third column channel.
**QRS AP_max_:** We calculated the sum of the maximum positive amplitude and maximum negative amplitude across all channels during the QRS complex, which was named QRS AP_max_.
**QRS interval:** We measured the QRS complex duration from the beginning of the QRS complex, which can be marked by the onset of the R wave or Q wave, extending to the termination of the S wave.
**T area, QRS area, and QRS-T area:** QRS amplitude and duration could be combined through curve integration. Referring to the work of Milla Karvonen and Silvia Comani (26, 27), we defined the QRS area as the sum of the absolute values of the areas under the QRS curve. The T area was obtained in the same way, and the QRS-T area was defined as the difference between these two.
**QRS product:** For ease of daily use, we also introduced the parameter QRS product, which directly multiplied QRS AP_max_ by QRS interval.
**DM_50_:** Based on the approach used by M Nomura's research (25), we also introduced the moving dipole technique for assessing the electromotive force augmentation in individuals with LVH, and measured the dipole moment 50 ms after the start of QRS depolarization.
**TT-MA and QRS-T angle:** To reflect the dynamic changes in the repolarization period, we selected two MFM parameters. The mean angle of the magnetic vector as it traverses from the positive to the negative pole during the T wave was represented as TT-MA, while the QRS-T angle denotes the variance in the average magnetic orientation between the T wave and the QRS complex.

### Statistical analysis

All statistical analyses were performed in SPSS version 23.0 (IBM Corp., Armonk, NY, USA) and R version 4.2.2. A *p*-value < 0.05 indicated statistical significance. Continuous variables were reported as mean ± standard deviation and analyzed with Mann–Whitney tests or t tests for groups comparisons. Categorical variables were described by frequency (percentage) and analyzed with *χ*^2^ test for groups comparisons. The statistical method of circular distribution was used for angle values, and Watson-William was used to test the difference of angle change between the two groups. MCG parameters with a *p*-value <0.05 were included in the multivariate regression analysis and a stepwise forward method was used to select the significant variables. A Nomogram was constructed to estimate the probability of LVH. The model's discriminative ability was evaluated using the area under the curve (AUC) of the receiver operating characteristic curve. Calibration Curves were evaluated graphically by plotting the difference between the model's predicted outcome risks and the actual observed frequencies. The net reclassification index (NRI) was used to analyze the improvement of the classification performance of the MCG model compared with ECG.

## Results

The retrospective cohort included 337 individuals, of which 39.2% were female. According to echocardiography indicators, patients were divided into non-LVH group and LVH group. Table 1 shows the demographic characteristics of these subjects. The proportion of female patients in the non-LVH group was higher than that in the LVH group either in the retrospective cohort or in the prospective cohort. The BMI of the LVH group was significantly higher than that of the non-LVH group in the retrospective cohorts (*P* < 0.05). Statistical analysis indicated no substantial differences in age (*P* > 0.05) between the two groups. However, the prevalence of hypertension in the LVH group was significantly higher than that in the non-LVH group [80.4% (*n* = 78) vs. 54.6% (*n* = 131), *P* < 0.001]. Levels of creatine kinase-MB (CK-MB), troponin I (TnI) and proBNP were markedly lower in the non-LVH group than those in the LVH group (*P* < 0.001). The level of HDL was higher in the non-LVH group than in the LVH group (*P* = 0.001). Baseline Echocardiography parameters including LVWT and LVMI in the LVH group were larger than those observed in the non-LVH group (13.07 ± 2.6, 118.28 ± 27.96 vs. 8.78 ± 0.87, 64.08 ± 11.46, *P* < 0.001). However, the LVEF was reduced as opposed to the non-LVH group (59.33 ± 8.34 vs. 64.28 ± 3.77, *P* < 0.001).

**Table 1:** Demographic characteristics and clinical information of subjects (*n* = 481).

Characteristic	Retrospective cohort (*n* = 337)			Prospective cohort (*n* = 144)		
	LVH group (*n* = 97)	Non-LVH group (*n* = 240)	*p*-value	LVH group (*n* = 55)	Non-LVH group (*n* = 89)	*p*-value
Gender = Female	38 (39.2%)	124 (51.7%)	0.05	18 (32.7)	47 (52.8)	0.029
Age, yrs	57.96 (14.12)	60.08 (11.06)	0.19	61.60 (12.88)	60.66 (10.35)	0.406
BMI, kg/m^2^	25.27 (4.48)	23.99 (2.94)	0.009	24.90 (4.00)	23.79 (3.06)	0.129
Hypertension	78 (80.4%)	131 (54.6%)	<0.001	48 (87.3)	42 (47.2)	<0.001
Diabetes	30 (30.9%)	49 (20.4%)	0.06	24 (43.6)	18 (20.2)	0.005
Smoking	6 (6.2%)	31 (12.9%)	0.11	16 (29.1)	12 (13.5)	0.037
Troponin I, μg/L	0.09 (0.33)	0.02 (0.09)	<0.001	0.38 (1.36)	0.00 (0.01)	<0.001
CK-MB, U/L	4.43 (4.76)	1.86 (2.11)	<0.001	5.60 (13.33)	1.67 (1.49)	0.011
proBNP, pg/ml^a^	938.00 [176.00,5,370.00]	43.95 [20.00,93.28]	<0.001	129.00 [34.05, 467.00]	38.70 [22.60, 80.00]	<0.001
Total cholesterol, mmol/L	4.27 (1.10)	4.34 (0.98)	0.41	4.00 (1.29)	4.46 (1.13)	0.021
Triglycerides, mmol/L	1.51 (1.03)	1.44 (0.91)	0.26	1.76 (1.51)	1.37 (0.90)	0.087
HDL, mmol/L	1.11 (0.35)	1.23 (0.35)	0.001	1.08 (0.33)	1.25 (0.33)	0.002
LDL, mmol/L	2.49 (0.90)	2.50 (0.78)	0.62	2.27 (0.98)	2.59 (0.93)	0.061
Echocardiography						
LVWT, mm	13.07 (2.60)	8.78 (0.87)	<0.001	13.43 (2.01)	8.65 (0.89)	<0.001
LVMI, g/m^2^	118.28 (27.96)	64.08 (11.46)	<0.001	100.56 (28.56)	71.15 (12.99)	<0.001
LVEF, %	59.33 (8.34)	64.28 (3.77)	<0.001	62.76 (6.11)	64.89 (3.00)	0.025

Data are presented as mean ± SD or *n* (%).

^a^ Presented as median [interquartile range].

LVH, left ventricular hypertrophy; BMI, body mass index; CK-MB, creatinine kinase-MB isoenzyme; proBNP, pro-brain natriuretic peptide; HDL, high-density lipoprotein; LDL, low-density lipoprotein; LVWT, left ventricular wall thickness; LVMI, left ventricular mass index; LVEF, left ventricular ejection fraction.

The prospective cohort consisted of 55 individuals with LVH and 89 without it. As presented in Table 1, apart from BMI, the prevalence of diabetes, smoking and total cholesterol, we discovered that the demographic characteristics, clinical baseline, laboratory and ultrasound characteristics of the prospective cohort were comparable to those of the retrospective cohort data.

**Figure 3:**
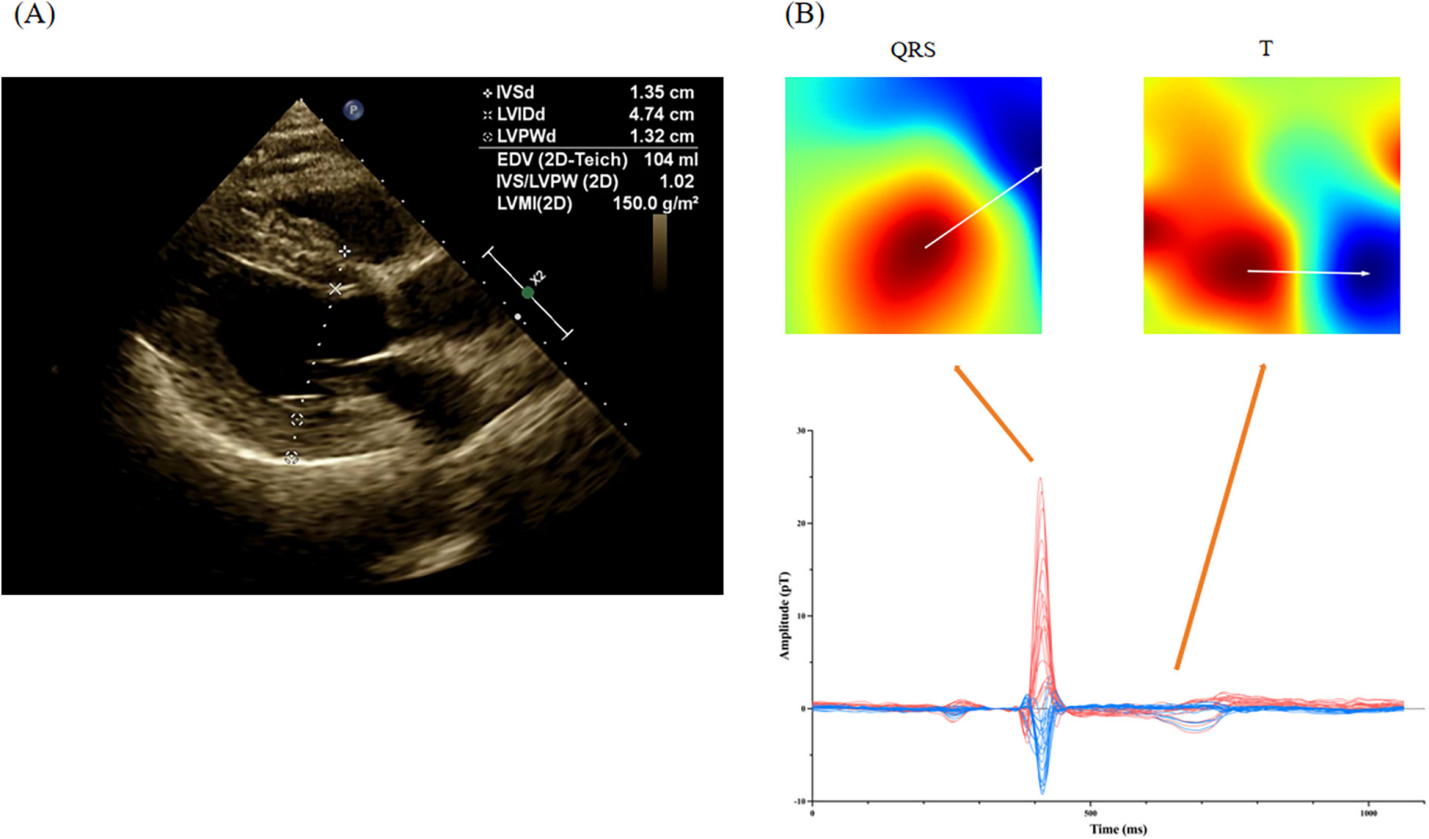
Representative MCG manifestation for a LVH patient. **(A)** The echocardiography of a LVH patient with hypertension; **(B)** the magnetocardiography of this patient. In a patient diagnosed with hypertension, echocardiography revealed thickening of the left ventricular posterior wall and the interventricular septum, approximately 13 mm. Magnetocardiography demonstrated a substantial rise in the amplitude of the QRS complex, alongside a notable increase in the QRS area. Additionally, T-wave magnetic fields were disordered, indicating alterations in magnetic field direction. The specific parameters recorded were as follows: QRS APmax = 34.2 pT, S_B5_ + R_D3_ = 27.4 pT, QRS area = 216.7 pT*ms, TT-MA = 356.4°, and QRS-T angle = 40.1°.

We analyzed MCG parameters in the two groups in the retrospective cohort and found 9 parameters with statistically significant differences, as shown in Table 2. SB5 + RD3, QRS APmax, QRS product, T area,QRS area, QRS-T area, DM50 and QRS-T angle exhibited higher in the LVH group than those in the non-LVH group (18.47 ± 12.42 vs. 14.34 ± 8.95, *P* = 0.001; 27.82 ± 17.50 vs. 17.56 ± 10.52, *P* < 0.001; 2,472.06 ± 1,679.29 vs. 1,520.46 ± 1,046.50, *P* < 0.001; 185.95 ± 125.15 vs. 105.12 ± 62.48, *P* < 0.001; 123.95 ± 84.69 vs. 101.49 ± 64.33, *P* = 0.025; 62.00 ± 88.05 vs. 3.63 ± 44.50, *P* < 0.001; 416.39 ± 842.29 vs. 133.88 ± 399.75, *P* < 0.001; 74.69 ± 53.61 vs. 38.17 ± 36.65, *P* < 0.001), and TT-MA in the LVH group was significantly lower (18.20 ± 32.44 vs. 60.14 ± 52.22, *P* < 0.001). The typical manifestation of these MCG parameters in an LVH patient diagnosed with hypertension is presented in Figure 3.

**Table 2:** Effectiveness of MCG parameters in the retrospective cohort.

Parameters	LVH group (*n* = 97)	Non-LVH group (*n* = 240)	*P*-value
S_B5_ + R_D3_, pT	18.47 ± 12.42	14.34 ± 8.95	0.001
QRS AP_max_, pT	27.82 ± 17.50	17.56 ± 10.52	<0.001
QRS interval, ms	86.90 ± 14.36	84.25 ± 10.91	0.212
QRS product, pT*ms	2,472.06 ± 1,679.29	1,520.46 ± 1,046.50	<0.001
QRS area, pT*ms	185.95 ± 125.15	105.12 ± 62.48	<0.001
T area, pT*ms	123.95 ± 84.69	101.49 ± 64.33	0.025
QRS-T area, pT*ms	62.00 ± 88.05	3.63 ± 44.50	<0.001
DM_50_, 10^−6^ Am	416.39 ± 842.29	133.88 ± 399.75	<0.001
TT-MA,°	18.20 ± 32.44	60.14 ± 52.22	<0.001
QRS-T angle,°	74.69 ± 53.61	38.17 ± 36.65	<0.001

Data are presented as mean ± SD.

**Figure 4:**
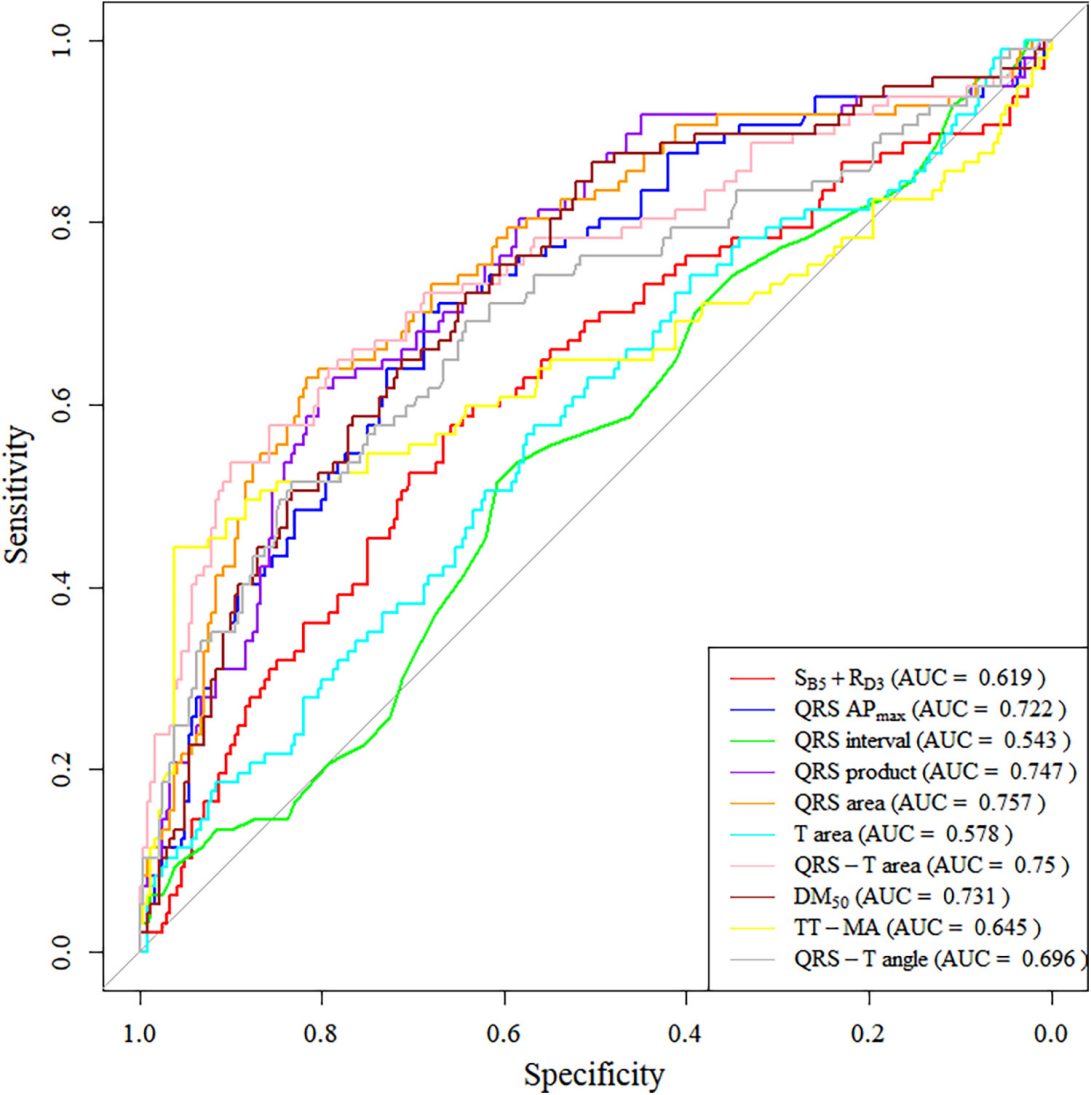
ROC curve for MCG parameters in the retrospective cohort.

We also plotted ROC curves for these parameters (Figure 4), with 5 parameters having an AUC greater than 0.7 (QRS AP_max_, QRS product, QRS area, QRS-T area, DM_50_). Specifically, the sensitivity, specificity, and AUC for using QRS-area to diagnose LVH were 62.9%, 81.7%, and 0.757, respectively; for the QRS product, the sensitivity was 67%, the specificity was 71.3%, and the AUC was 0.747.

**Figure 5:**
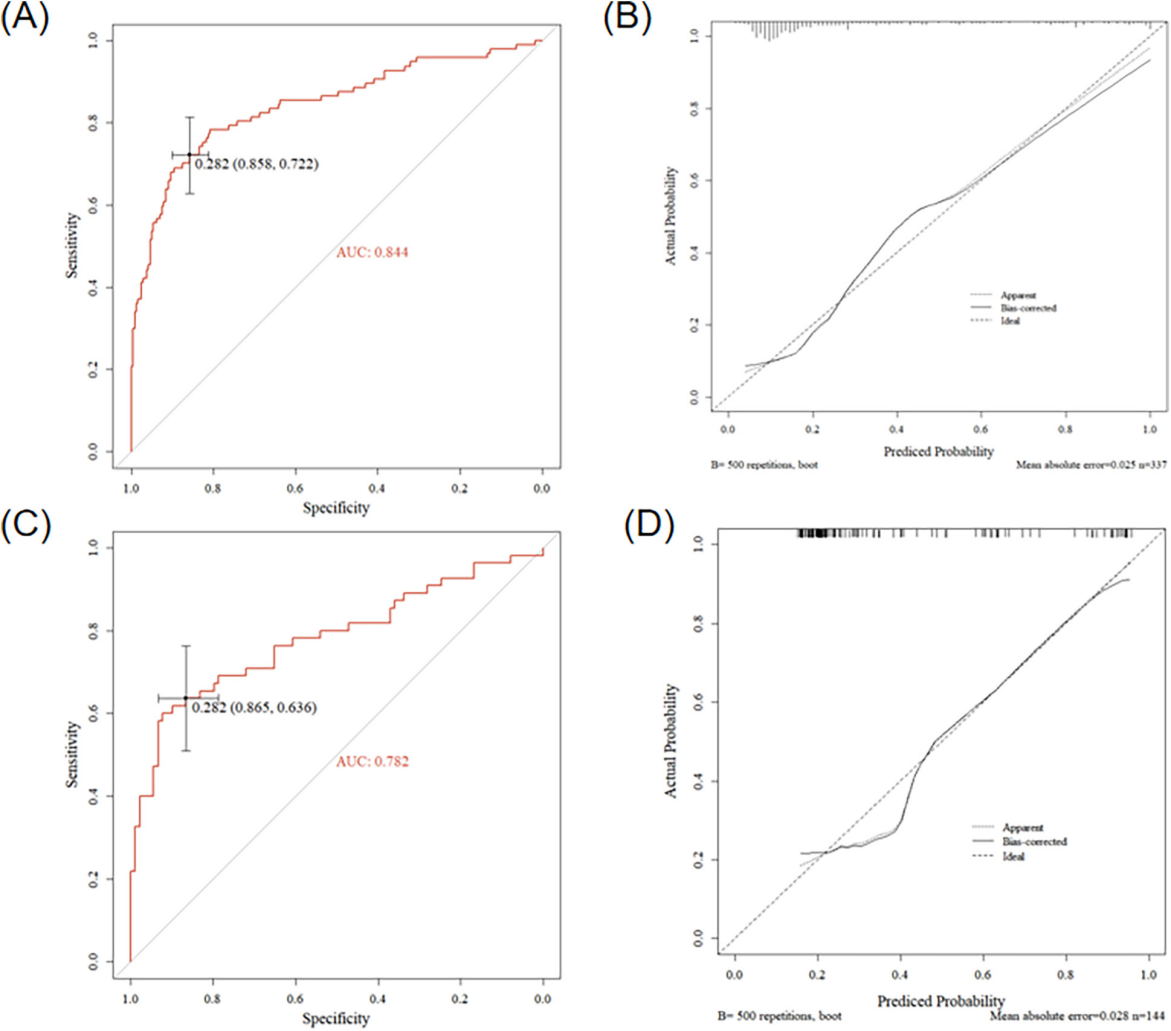
Results of the MCG model in the retrospective cohort and the prospective cohort. **(A)** ROC curve of the model in the retrospective cohort, **(B)** Calibration curve of the model in the retrospective cohort, **(C)** ROC curve of the model in the prospective cohort, **(D)** Calibration curve of the model in the prospective cohort.

**Figure 6:**
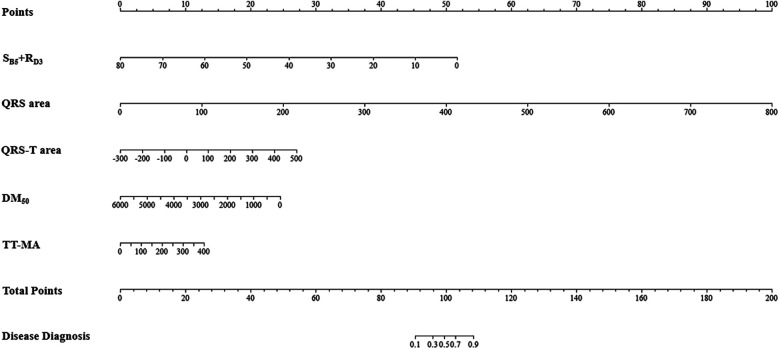
Nomogram of the MCG-LVH model. For example, within the MCG-LVH model, a patient with S_B5_ + R_D3_ = 10 (Points = 45), QRS area = 100 (Points = 12), QRS-T area = 400 (Points = 24), DM_50_ = 500 (Points = 23) and TT-MA = 50 (Points = 2), the total score of the indicator is 45 + 12 + 24 + 23 + 2 = 106. Consequently, the predicted probability of suffering from LVH for this patient is 81.0%.

Using forward stepwise regression, we selected variables from the statistically significant parameters, and 5 variables were finally included in the model (S_B5_ + R_D3_, QRS area, QRS-T area, DM_50_, TT_MA), as presented in Table 3. The MCG-LVH model had a sensitivity of 72.2%, specificity of 85.8%, PPV of 67.3%, NPV of 88.4%, accuracy of 81.9% and AUC of 0.844. We further validated the model's accuracy using the prospective cohort. In the validation set, the model's sensitivity was 63.6%, specificity was 86.5%, PPV was 74.5%, NPV was 79.4%, accuracy was 77.8% and AUC was 0.782, as presented in Table 4. The validation set's calibration curve also demonstrated consistency between the ideal curve and the calibration curve, as illustrated in Figure 5. A nomogram was drawn to assess the risk of LVH, where higher total points, derived from the aggregate points for all predictors, signified a greater risk of LVH (Figure 6).

**Table 3:** Multivariate logistics regression in the retrospective cohort (forward conditional).

Variables	*β*	OR	95% CI	*P*-value
S_B5_ + R_D3_	−0.159	0.853	(0.797–0.913)	<0.001
QRS area	0.031	1.031	(1.02–1.042)	<0.001
QRS-T area	0.008	1.008	(1.002–1.015)	0.01
DM_50_	−0.001	0.999	(0.998–1.000)	0.026
TT-MA	0.008	1.008	(1.005–1.011)	<0.001
Intercept	−3.191	0.041		<0.001

The formula of the model: logit *P* = −3.191 + (−0.159 * S_B5_ + R_D3_) + 0.031* QRS area + 0.008 * QRS-T area + (−0.001 * DM_50_) + 0.008 * TT-MA.

*P* = e^logit *P*^/(1 + e^logit *P*^), *P* means predicted probability.

**Table 4:** Diagnostic performance of the MCG model in the retrospective cohort and the prospective cohort.

Model	Retrospective cohort	Prospective cohort
	(*n* = 337)	(*n* = 144)
Cut-off value	0.282	0.282
AUC (95% CI)	0.844 (0.792–0.895)	0.782 (0.697–0.866)
*P*-value	<0.001	<0.001
Sensitivity (95% CI)	72.2% (62.0–80.6)	63.6% (49.5–75.9)
Specificity (95% CI)	85.8% (80.6–89.9)	86.5% (77.2–92.5)
Positive Predictive Value (95% CI)	67.3% (57.3–76.0)	74.5% (59.4–85.6)
Negative Predictive Value (95% CI)	88.4% (83.4–92.1)	79.4% (69.7–86.7)
Accuracy (95% CI)	81.9% (77.4–85.9)	77.8% (70.1–0.84.3)

We utilized our model and the clinical diagnostic criteria of ECG to analyze the results of the two modalities separately, subjects involved were the 469 patients mentioned above (12 subjects were excluded due to missing results of ECG). Our analysis demonstrated that MCG exhibits superior sensitivity and diagnostic accuracy compared to ECG (Sensitivity: 69.9% vs. 52.4%, Accuracy: 81.0% vs. 76.1%) while maintaining comparable specificity between the two modalities (Specificity: 85.9% vs. 86.5%). A comprehensive summary of these findings is presented in Table 5. It was shown that the predictive efficiency of the MCG model was 18.1% higher than that of ECG in terms of the proportion of correct classification in the Net Reclassification Index (NRI) analysis.

**Table 5:** Diagnostic performance of ECG and MCG in the study population.

Echocardiography	ECG		MCG	
	(+)	(−)	(+)	(−)
(+)	75	68	100	43
(−)	44	282	46	280
Sensitivity (95% CI)	52.4% (44.0–60.8)		69.9% (61.6–77.2)	
Specificity (95% CI)	86.5% (82.2–89.9)		85.9% (81.5–89.4)	
Positive Predictive Value (95% CI)	63.0% (53.6–71.6)		68.5% (60.2–75.8)	
Negative Predictive Value (95% CI)	80.6% (76.0–84.5)		86.7% (82.4–90.1)	
Accuracy (95% CI)	76.1% (72.0 −79.9)		81.0% (77.2–84.5)	
Net reclassification index	Reference		18.1%	
*P*-value	<0.001				

## Discussion

In our study, MCG parameters including S_B5_ + R_D3_, QRS AP_max_, QRS product, T area,QRS area, QRS-T area, DM_50,_ QRS-T angle and TT-MA were found to have significant predictive value for LVH. Diagnosis model for diagnosing LVH was established by combing five MCG indicators (S_B5_ + R_D3_, QRS area, QRS-T area, DM_50_, TT_MA), and the satisfactory diagnostic performance was verified in the prospective cohort.

Previous research (24–27) rerolled patients with simple hypertensive LVH or simple HCM, and the number of cases was small. Our study subjects encompassed patients with LVH caused by various reasons (HCM, hypertensive heart disease, uremic cardiomyopathy and so on), including different degrees of hypertrophy and various conditions of cardiac function, which broadened the study population compared to previous research. Levels of TnI, pro-BNP and CK-MB in the LVH group were higher than in the control group, suggesting that the LVH group exhibited more pronounced myocardial injury and cardiac dysfunction than the non-LVH group. LVWT, LVMI and LVEF on echocardiography showed notable disparities between the two groups both in the retrospective study and the prospective study.

Fujino et al. (24) found that if S_B3_ + R_E3_ > 4 × 10^−7^ T, it was indicated as left ventricular overload. Using echocardiography as the gold standard, the sensitivity of ECG and MCG were 48.6% and 44.3%, respectively. Due to differences in device structure and signal acquisition range, we chose to use S_B5_ + R_D3_ in MCG to evaluate the QRS amplitude. Our experimental results illustrated that the sensitivity and specificity of this index in detecting LVH were highly improved. The hypertrophy of cardiac muscle fibers, leads to an increased cross-sectional area and a greater number of electrical dipoles during depolarization, resulting in an augmented depolarization vector. S_B5_ + R_D3_ and QRS AP_max_ reflected these changes in the left ventricular depolarization vector to some extent.

The thickening of the ventricular wall, coupled with prolonged conduction time from the endocardial to epicardial layers due to myocardial cell degeneration, extends the duration of ventricular depolarization and broadens the QRS complex interval. Considering that using QRS interval alone was not specific for diagnosing LVH because other conditions like bundle branch block could also lead to QRS prolongation, we combined QRS amplitude and duration through curve integration. Referring to Milla Karvonen and Comani S's research, we introduced QRS product, QRS area, and QRS-T area into our study (26, 27). Our experiment not only proved that they could detect left ventricular hypertrophy but also had high sensitivity and specificity.

Nomura M et al. (25) demonstrated that the moving dipole method proved effective in assessing the heightened electromotive force in individuals with left ventricular overload. The value of DM_50_ was also verified in our study.

Ventricular wall thickening could be accompanied by myocardial ischemia (resulting in abnormal repolarization), manifesting as ST-T segment abnormalities on ECG. Although ST-T changes could appear in multiple heterogeneous diseases, such as myocardial ischemia or electrolyte disturbance, they could significantly improve the sensitivity of LVH diagnosis when used as complementary indicators in a combined diagnostic model. To reflect the dynamic magnetic changes in the repolarization period, we selected two MFM parameters (QRS-T angle and TT-MA) (30). These two parameters in MFM were not presented in previous MCG studies to detect LVH.

Our results suggested that single MCG parameters alone could achieve good diagnostic performance for LVH, but the diagnostic performance of the MCG-LVH model combined with these parameters (SB5 + RD3, QRS area, QRS-T area, DM50, TT_MA) was the best, superior to the MCG parameter alone. The sensitivity and specificity of the MCG-LVH model in the retrospective study were 72.2% and 85.8%, and 63.6% and 86.5% in the prospective study. The sensitivity was probably lower in the prospective study because many of the cases had milder left ventricular overloading than those in the retrospective study. We not only measured the diagnostic efficacy of MCG-LVH but also compared it with that of ECG-LVH criteria. The specificity of ECG (86.5%) and MCG (85.9%) were almost equal, but the MCG-LVH demonstrated significantly higher sensitivity and accuracy compared to the ECG (Sensitivity: 69.9% vs. 52.4%, Accuracy: 81.0% vs. 76.1%).

Compared with previous studies, the diagnostic efficacy of MCG in our study is better, which may be ascribed to the following reasons: (1) Magnetic field measurement is unaffected by peripheral tissues, penetrating high-impedance materials without distortions (4). (2) MCG is more sensitive to tangential currents of hypertrophic cardiomyocytes and can detect both direct and alternating current signals, providing a sharper perception of body surface potential differences. (3) With the continuous progress of magnetic field detection sensors and further reduced signal-to-noise ratio, we can obtain higher MCG quality to provide more accurate information for clinical use. (4) The comprehensive indicators of the MCG-LVH model selected in our study are more comprehensive and accurate.

Echocardiography is capable of measuring the anatomical structures of the heart, including the thickness of the left ventricular wall, the mass of the left ventricle, and the ejection fraction (EF) as an indicator of cardiac function. However, the accuracy of linear measurement methods is limited in cases such as asymmetric hypertrophy and left ventricular enlargement. MCG can detect electrophysiological changes associated with cardiomyocyte remodeling in left ventricular hypertrophy (LVH), which further corroborates the findings of our model, achieving a sensitivity of 72.2% and a specificity of 85.8%. It should be noted that not all patients experience localized cellular damage due to insufficient blood supply to the hypertrophic myocardium. Conversely, not all patients with MCG-detected abnormalities exhibit myocardial hypertrophy. MCG can detect changes in electrical activity caused by LVH or myocardial ischemia resulting from hypertrophy, which is beyond the reach of echocardiography. Both methods are not competing but rather complementary to each other.

MCG also has many other advantages, such as the lack of necessity for specific training in device operation, as well as no requirement under physician oversight. This study further confirmed that MCG could be utilized to detect left ventricular hypertrophy using identified 5 MCG parameters, thus, MCG might simultaneously provide both classic clinical information on ischemia and additional information on LVH, expanding the popularization of MCG in clinical practice in the future. The validity and generalizability of this MCG-LVH model require confirmation through large-scale, multi-center clinical trials, which will also constitute the focus of our future research endeavors.

## Conclusions

This study indicates that 5 MCG parameters could be used to detect LVH, expanding the clinical application of MCG besides the early diagnosis of myocardial ischemia.

## Data Availability

The datasets presented in this study can be found in online repositories. The names of the repository/repositories and accession number(s) can be found in the article/Supplementary Material.
